# Interrupted time-series analysis of active case-finding for tuberculosis during the COVID-19 pandemic, Zambia

**DOI:** 10.2471/BLT.21.286109

**Published:** 2022-01-25

**Authors:** Patrick S Lungu, Andrew D Kerkhoff, Monde Muyoyeta, Clara C Kasapo, Sarah Nyangu, Mary Kagujje, Rhehab Chimzizi, Sulani Nyimbili, Morton Khunga, Nancy Kasese-Chanda, Victoria Musonda, Bushimbwa Tambatamba, Christopher M Kombe, Charles Sakulanda, Kizito Sampa, Andrew Silumesii, Kennedy Malama

**Affiliations:** a National Tuberculosis and Leprosy Control Programme, Ministry of Health, Ndeke House, Haile-Selaise Road, PO Box 30205, Lusaka, Zambia.; b Division of HIV, Infectious Diseases and Global Medicine, University of California San Francisco, San Francisco, United States of America.; c Centre for Infectious Disease Research in Zambia, Lusaka, Zambia.; d United States Agency for International Development, Lusaka, Zambia.; e Eradicate Tuberculosis Project, United States Agency for International Development, Lusaka, Zambia.; f Ministry of Health, Lusaka, Zambia.

## Abstract

**Objective:**

To evaluate the impact of the coronavirus disease 2019 (COVID-19) pandemic and the subsequent implementation of tuberculosis response measures on tuberculosis notifications in Zambia.

**Methods:**

We used an interrupted time-series design to compare monthly tuberculosis notifications in Zambia before the pandemic (January 2019 to February 2020), after implementation of national pandemic mitigation measures (April 2020 to June 2020) and after response measures to improve tuberculosis detection (August 2020 to September 2021). The tuberculosis response included enhanced data surveillance, facility-based active case-finding and activities to generate demand for services. We used nationally aggregated, facility-level tuberculosis notification data for the analysis.

**Findings:**

Pre-pandemic tuberculosis case notifications rose steadily from 2890 in January 2019 to 3337 in February 2020. After the start of the pandemic and mitigation measures, there was a −22% (95% confidence interval, CI: −24 to −19) immediate decline in notifications in April 2020. Larger immediate declines in notifications were seen among human immunodeficiency virus (HIV)-positive compared with HIV-negative individuals (−36%; 95% CI: −38 to −35; versus −12%; 95% CI: −17 to −6). Following roll-out of tuberculosis response measures in July 2020, notifications immediately increased by 45% (95% CI: 38 to 51) nationally and across all subgroups and provinces. The trend in notifications remained stable through September 2021, with similar numbers to the predicted number had the pandemic not occurred.

**Conclusion:**

Implementation of a coordinated public health response including active tuberculosis case-finding was associated with reversal of the adverse impact of the pandemic and mitigation measures. The gains were sustained throughout subsequent waves of the pandemic.

## Résumé

**Objectif:**

Évaluer l'impact de la pandémie de maladie à coronavirus 2019 (COVID-19) et la mise en œuvre subséquente de mesures d'intervention pour la notification des cas de tuberculose en Zambie.

**Méthodes:**

Nous avons utilisé un modèle de série chronologique interrompue pour comparer les cas de tuberculose signalés chaque mois en Zambie avant la pandémie (de janvier 2019 à février 2020), après le déploiement de mesures visant à atténuer les effets de la pandémie à l'échelle nationale (d'avril 2020 à juin 2020) et après l'adoption de mesures d'intervention destinées à améliorer le dépistage de la tuberculose (d'août 2020 à septembre 2021). Parmi les mesures d'intervention contre la tuberculose figuraient une surveillance accrue des données, un dépistage actif des cas au sein des établissements, ainsi que des activités créant une demande de services. Notre analyse s'est fondée sur des données de notification de la tuberculose récoltées dans les établissements et regroupées pour l'ensemble du pays.

**Résultats:**

Avant la pandémie, le nombre de cas de tuberculose signalés a connu une augmentation constante, passant de 2890 en janvier 2019 á 3337 en février 2020. Après le début de la pandémie et l'instauration de mesures cherchant à en atténuer les effets, le nombre de notifications a instantanément baissé de 22% (IC de 95%: −24 à −19) en avril 2020. Les individus positifs au virus de l'immunodéficience humaine (VIH) affichaient une diminution immédiate des notifications plus importante que les individus négatifs au VIH (−36%; IC de 95%: −38 à −35; contre −12%; IC de 95%: −17 à −6). À la suite du déploiement des mesures d'intervention contre la tuberculose en juillet 2020, les notifications ont bondi de 45% (IC de 95%: 38 à 51), tant au niveau national qu'au sein des sous-groupes et provinces. Cette tendance s'est poursuivie tout au long du mois de septembre 2021, avec des chiffres similaires aux prévisions attendues en l'absence de pandémie.

**Conclusion:**

La mise en œuvre d'une intervention de santé publique coordonnée impliquant notamment un dépistage actif de la tuberculose a permis de limiter l'impact négatif de la pandémie et des mesures visant à en atténuer les effets. Les bénéfices de cette intervention ont résisté aux vagues successives de la pandémie.

## Resumen

**Objetivo:**

Evaluar los efectos de la pandemia de la enfermedad por coronavirus 2019 (COVID-19) y la posterior aplicación de las medidas de respuesta a la tuberculosis en las notificaciones de esta enfermedad en Zambia.

**Métodos:**

Se empleó un diseño de series temporales interrumpidas para comparar las notificaciones mensuales de tuberculosis en Zambia antes de la pandemia (enero de 2019 a febrero de 2020), después de la aplicación de las medidas nacionales de mitigación de la pandemia (abril de 2020 a junio de 2020) y después de las medidas de respuesta para mejorar la detección de la tuberculosis (agosto de 2020 a septiembre de 2021). La respuesta a la tuberculosis incluyó la mejora de la vigilancia de los datos, la búsqueda activa de casos en los centros y las actividades para generar demanda de servicios. Para el análisis se utilizaron datos de notificación de tuberculosis agregados a nivel nacional y a nivel de los centros.

**Resultados:**

Las notificaciones de casos de tuberculosis anteriores a la pandemia aumentaron de manera constante de 2890 en enero de 2019 a 3337 en febrero de 2020. Tras el inicio de la pandemia y las medidas de mitigación, se produjo un descenso inmediato del -22 % (IC del 95 %: -24 a -19) en las notificaciones en abril de 2020. Se observaron mayores descensos inmediatos en las notificaciones entre las personas infectadas por el virus de la inmunodeficiencia humana (VIH) en comparación con las personas no infectadas (-36 %; IC del 95 %: -38 a -35; frente a -12 %; IC del 95%: -17 a -6). Después del despliegue de las medidas de respuesta a la tuberculosis en julio de 2020, las notificaciones aumentaron de inmediato en un 45 % (IC del 95 %: 38 a 51) a nivel nacional y en todos los subgrupos y provincias. La tendencia de las notificaciones se mantuvo estable hasta septiembre de 2021, con cifras similares a las previstas si la pandemia no hubiera ocurrido.

**Conclusión:**

La aplicación de una respuesta de salud pública coordinada que incluyera la búsqueda activa de los casos de tuberculosis se asoció a la reducción de los efectos negativos de la pandemia y a las medidas de mitigación. Los beneficios se mantuvieron durante las siguientes oleadas de la pandemia

## ملخص

**الغرض:**

تقييم تأثير جائحة فيروس كورونا 2019 (كوفيد 19)، والتنفيذ اللاحق لإجراءات الاستجابة لمرض السل بعد تلقي إخطارات السل في زامبيا.

**الطريقة:**

لقد استخدمنا تصميم السلاسل الزمنية المتقطعة لمقارنة الإخطارات الشهرية عن السل في زامبيا قبل الجائحة (يناير/كانون ثاني 2019 إلى فبراير/شباط 2020)، وبعد تنفيذ الإجراءات الوطنية للتخفيف من الجائحة (أبريل/نيسان 2020 إلى يونيو/حزيران 2020)، وبعد إجراءات الاستجابة لتحسين اكتشاف السل (أغسطس/آب 2020 إلى سبتمبر/أيلول 2021). تضمنت الاستجابة لمرض السل تعزيز رصد البيانات، واكتشاف الحالات النشط في المرافق، وأنشطة لتوجيه الطلب على الخدمات. استخدمنا بيانات إخطارات السل المجمعة على مستوى المرافق، وعلى الصعيد الوطني، من أجل التحليل.

**النتائج:**

ارتفعت إخطارات السل لما قبل لجائحة بشكل ثابت من 2890 في يناير/كانون ثاني 2019 إلى 3337 في فبراير/شباط 2020. بعد بدء الجائحة وإجراءات التخفيف، كان هناك انخفاض فوري بنسبة -22% (بفاصل ثقة مقداره 95%: -24 إلى -19) في الإخطارات في أبريل/نيسان 2020. وتمت ملاحظة انخفاضات فورية أكبر في الإخطارات بين الأفراد المصابين بفيروس نقص المناعة البشرية، عنها في الأفراد غير المصابين بفيروس نقص المناعة البشرية (-36%؛ بفاصل ثقة مقداره 95%: -38 إلى -35؛ مقابل -12%؛ بفاصل ثقة مقداره 95%: -17 إلى -6). بعد بدء تطبيق إجراءات الاستجابة للسل في يوليو/تموز 2020، زادت الإخطارات بشكل فوري بنسبة 45% (فاصل الثقة مقداره 95%: 38 إلى 51) على الصعيد الوطني وعبر جميع المجموعات الفرعية والمقاطعات. ظل الاتجاه في الإخطارات مستقرًا حتى سبتمبر/أيلول 2021، مع أرقام مشابهة للرقم المتوقع لو لم تقع الجائحة.

**الاستنتاج:**

إن تنفيذ الاستجابة المنسقة للصحة العمومية، بما في ذلك اكتشاف حالات السل النشط، ارتبط بانعكاس التأثير الضار للجائحة وإجراءات التخفيف. واستمرت المكاسب خلال الموجات اللاحقة للجائحة.

## 摘要

**目的:**

旨在评估赞比亚新型冠状病毒肺炎 (COVID-19) 疫情的影响以及后续结核病通报方面的结核病应对措施的实施情况。

**方法:**

我们采用中断时间序列设计来比较赞比亚每个月的结核病通报（从 2019 年 1 月至 2020 年 2 月）并且采用后续应对措施来提升结核病的检测（从 2020 年 8 月至 2021 年 9 月）。结核病应对措施包括增强数据监测、基于机构的主动病例发现和促进服务需求的活动。我们采用基于机构水平的全国性综合结核病通报数据来开展分析。

**结果:**

疫情前的结核病病例通报从 2019 年 1 月的 2890 例增长到 2020 年 2 月的 3337 例。在疫情爆发和采取管控措施之后，通报数在 2020 年 4 月立即减少了-22%（95% CI: -24 至-19）。与人体免疫缺损病毒 (HIV) 阴性相比，HIV 阳性的通报病例数立即减少的幅度更大（-36%; 95% CI: -38 至 -35；对比-12%; 95% CI: -17 至-6）。在 2020 年 7 月推出结核病应对措施之后，全国范围内以及各个地区和省份的通报数立即增加 45%（95% CI: 38 至 51）。截至 2021 年 9 月，通报的趋势保持稳定，与疫情发生前的预测数值相似。

**结论:**

包括结核病主动病例发现在内统筹部署的公共卫生应对措施的实施有助于消除疫情和管控措施的影响。在后续几轮疫情反复中，上述成果一直保持。

## Резюме

**Цель:**

Оценить влияние пандемии коронавирусной инфекции 2019 года (COVID-19) и последующего осуществления мер по борьбе с туберкулезом на уведомления о заболевании туберкулезом в Замбии.

**Методы:**

Для совершенствования мер по борьбе с туберкулезом авторы использовали анализ прерванных временных рядов и сравнили ежемесячные уведомления о заболевании туберкулезом в Замбии до пандемии (с января 2019 г. по февраль 2020 г.), после реализации национальных мер по смягчению последствий пандемии (с апреля по июнь 2020 г.) и после осуществления мер реагирования для улучшения выявления туберкулеза (с августа 2020 г. по сентябрь 2021 г.). Меры по борьбе с туберкулезом состояли из усиленного контроля данных, активного выявления случаев заболевания в медицинских учреждениях и мероприятий для создания спроса на услуги. Для анализа авторы использовали общенациональные данные об уведомлениях о заболевании туберкулезом на уровне медицинских учреждений.

**Результаты:**

Число зарегистрированных случаев заболевания туберкулезом до пандемии неуклонно росло: с 2890 случаев в январе 2019 года до 3337 случаев в феврале 2020 года. После начала пандемии и принятия мер по смягчению последствий пандемии в апреле 2020 г. произошло внезапное снижение количества уведомлений на 22% (95%-й ДИ: с 24 до 19). Более значительное внезапное снижение количества уведомлений наблюдалось среди ВИЧ-положительных лиц по сравнению с ВИЧ-отрицательными (36%; 95%-й ДИ: с 38 до 35; против 12%; 95%-й ДИ: с 17 до 6). После развертывания мер по борьбе с туберкулезом в июле 2020 г. количество уведомлений сразу же увеличилось на 45% (95%-й ДИ: с 38 до 51) на национальном уровне и во всех подгруппах и провинциях. Тенденция в части уведомлений оставалась стабильной до сентября 2021 года, при этом цифры были близки к прогнозируемым в случае отсутствия пандемии.

**Вывод:**

Осуществление скоординированных ответных мер в системе здравоохранения, включая активное выявление случаев заболевания туберкулезом, было связано с обращением вспять неблагоприятного воздействия пандемии и принятием мер по смягчению последствий пандемии. Результаты сохранялись на протяжении последующих волн пандемии.

## Introduction

Since the beginning of the global coronavirus disease 2019 (COVID-19) pandemic in early 2020, there were fears that it would devastate fragile health systems in resource-limited settings and erase hard-fought gains for several public health priorities, including tuberculosis.^1^^–^^4^ As COVID-19 continued to spread throughout the world in 2020, tuberculosis service infrastructure and resources were diverted towards the pandemic response. Many vulnerable individuals with undiagnosed tuberculosis had difficulty using tuberculosis services due to limited access (clinic closures, health worker shortages or to avoid crowds), fear of contracting COVID-19 or stigma related to tuberculosis and COVID-19.^5^ In a survey of 567 tuberculosis health professionals from 64 low- and middle-income countries, 233 (41%) said it was much harder or impossible for tuberculosis patients to seek care at facilities since the start of the pandemic. Likewise, 162 (29%) of respondents said it was difficult or nearly impossible to provide tuberculosis diagnostic services.^6^

Zambia is a high tuberculosis burden country where tuberculosis is a leading cause of mortality, especially among people living with human immunodeficiency virus (HIV).^7^^,^^8^ Following the identification of the first two COVID-19 cases in Zambia on 18 March 2020, the government quickly implemented several public health measures to prevent and mitigate the spread of severe acute respiratory syndrome coronavirus 2, (the virus causing COVID-19). In line with early reports from other countries, preliminary data in Zambia showed large reductions in tuberculosis notifications following the pandemic and related transmission risk mitigation measures. To address the adverse effects of the pandemic on tuberculosis outcomes and services, the National Tuberculosis and Leprosy Programme worked with implementation partners to design and implement a series of measures to improve tuberculosis case detection. Strategies included an enhanced surveillance system with active case-finding and activities to generate demand for services.

As of December 2021, there were 210 195 confirmed COVID-19 cases and 3667 confirmed COVID-19-related deaths among the general population in Zambia.^9^ However, it remains unknown how tuberculosis notifications in Zambia were affected by the pandemic and how notifications may have changed following the roll-out of several targeted tuberculosis-related activities to mitigate its impact. Therefore, we undertook an interrupted time-series analysis of national tuberculosis notification data to evaluate the impact of the pandemic and subsequent tuberculosis response measures and assess whether effects differed among key subgroups and by province.

## Methods

We undertook a retrospective analysis of Zambia’s tuberculosis case notifications recorded and reported from January 2019 through September 2021. All individuals diagnosed with drug-susceptible tuberculosis and registered for treatment initiation (notified) were included in the analysis, regardless of age or tuberculosis type (that is, new or retreatment).

### Data sources

For the analysis we used nationally aggregated, routinely captured tuberculosis programme data supplied by the National Tuberculosis and Leprosy Programme. Every health facility in Zambia uses a paper-based system to document tuberculosis case notifications. Once an individual with tuberculosis starts treatment, their data are recorded in a facility-level register: demographic characteristics (including age, sex, HIV status), tuberculosis laboratory results (such as the Xpert® MTB/RIF assay or smear microscopy status), and diagnosis and treatment start dates. Every month, facilities send tuberculosis notification data to the district, where they are aggregated. Data are then sent to the province, where they are aggregated again before being sent to the National Tuberculosis and Leprosy Programme. Next, a monitoring and evaluation team reviews the data; any incomplete or irregular data are clarified and corrected before undergoing final national-level data aggregation. To illustrate the relationship with COVID-19 infections over the same period, we also used publicly available data on the daily number of COVID-19 cases reported in Zambia.^10^

This study was a population-level analysis without the use of any patient identifiers. The University of Zambia biomedical research ethics committee reviewed the study and classified it as exempt from human subjects’ research review.

### Setting

**Fig. 1:**
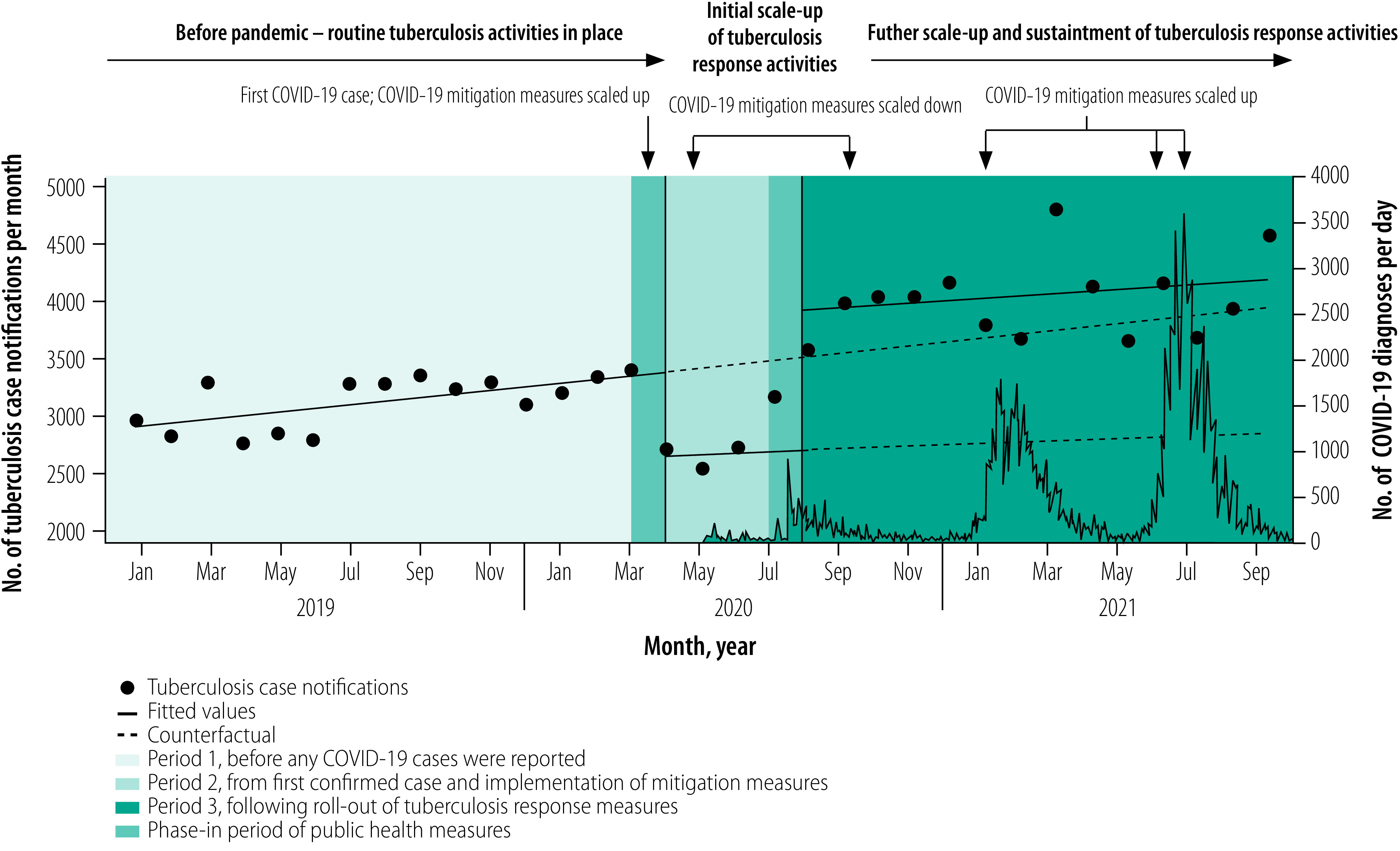
National level tuberculosis notifications in Zambia, January 2019 to September 2021

COVID-19: coronavirus disease 2019; HIV: human immunodeficiency virus.

Notes: Superimposed above the figure is a timeline showing when national COVID-19 transmission measures were scaled up and scaled down in response to initial and subsequent waves of COVID-19. The following mitigation measures were either scaled up or down.^11^ From 18–27 March 2020, the mitigation measures were scaled up: restaurants, bars and entertainment venues were closed; gatherings were limited to < 50 people; schools were closed; domestic and international travel was prohibited. On 16 April 2020 wearing masking was required in public. From 24 April–8 May 2020 restaurants, entertainment venues and places of worship reopened. On 25 June 2020 air travel resumed. Between 12 and 21 September schools reopened; bars and nightclubs reopened but with restrictions on capacity and hours of operation. On 6 January 2021 bars and nightclubs were limited to take-out only and public gatherings were suspended. On 26 May 2021 political rallies were suspended. On 18 June 2021 restaurants were limited to take-out only; numbers at funerals and weddings were limited; religious services were restricted; schools were closed. Between 5 and 16 August 2021 schools were gradually reopened.

Measures to mitigate the risk of COVID-19 transmission were implemented nationally in late March 2020. Mitigation measures comprised travel restrictions, closures of restaurants, bars and educational institutions, and limitations of public gatherings to less than 50 people. While many of these measures had been scaled down by September 2020, most individuals in Zambia resumed their normal daily activities by August 2020, in large part because they could not afford the direct and indirect costs of continued compliance with the measures. As the pandemic progressed, mitigation measures were scaled up and eased in response to subsequent surges in COVID-19 infections (Fig. 1).^11^

During the initial period in which pandemic mitigation measures were in place in Zambia, tuberculosis diagnostic and treatment services remained open throughout the country. Adaptations to existing tuberculosis services were made to protect vulnerable patients and staff and avoid overcrowding at facilities, including pausing community-based activities, providing prolonged tuberculosis medication refills, and undertaking telephone-based household contact tracing and tuberculosis treatment assessments. HIV services made similar adaptations. HIV services already provided extended multi-month dispensing of antiretroviral therapy (ART) for up to 6 months to clinically stable patients. This strategy was therefore substantially scaled up across Zambia at the onset of the pandemic.^12^

In July 2020, following 3 months of declining tuberculosis case notifications (April to June), the National Tuberculosis and Leprosy Programme worked with several stakeholders to design and begin implementing a multicomponent strategy to improve tuberculosis surveillance and tuberculosis detection during the ongoing COVID-19 pandemic (Box 1).

Box 1 **Activities implemented to mitigate the impact of COVID-19 on tuberculosis diagnosis and treatment services in Zambia**

**Tuberculosis situation room**

Each week, staff at the National Tuberculosis and Leprosy Programme held an online meeting to review national tuberculosis notification data. Participants were staff from implementing partner organizations and those involved in tuberculosis programming from the facility level through to the national level. Centralized teams and higher-performing facilities and districts (selected based on comparisons to set tuberculosis notification targets) were able to share best practices and provide support to lower-performing facilities and districts. To support this initiative, the National Tuberculosis and Leprosy Programme developed a weekly data reporting tool; built local capacity among facility-based health-care workers to improve record-keeping at all patient entry points; and began reporting data weekly rather than monthly. The permanent secretary of the health ministry officially launched this initiative to enhance support by leadership at each level. Since the initiative’s launch in July 2020, weekly meetings have been sustained throughout the pandemic.

**Facility-based active case-finding**

Beginning in July 2020, we designed a strategy to motivate public health facilities across all provinces to begin systematic symptoms-based screening of all individuals attending the facility for active tuberculosis disease, regardless of the reason for presentation. We initially prioritized facilities contributing 80% of notifications at the district level. Activities included (i) training and reorienting health-care staff at each facility using standard operating procedures and standardized training slides; (ii) increasing the availability of digital chest X-rays for use as a triage test; and (iii) improving access to urine tuberculosis tests (lateral flow urine lipoarabinomannan assay). Over the subsequent months, we scaled up the number of tuberculosis diagnostic facilities providing facility-based active case-finding. Once saturation was achieved at diagnostic facilities, we introduced systematic, symptoms-based screening at tuberculosis treatment-only facilities (those with no tuberculosis diagnostic capacity). Sputum samples were collected from patients who screened positive for tuberculosis and sent to local diagnostic facilities for microbiological testing. We gave technical supervision, support and mentorship around this initiative to individual health facilities through weekly provincial-level tuberculosis situation room meetings. Since initial scale-up, all facilities that we have trained have sustained systematic symptoms-based screening throughout the pandemic.

**Demand generation**

Initially, community-based volunteers were working within health facilities to generate demand for tuberculosis services among community members already attending health facilities for any reason. After initial COVID-19 mitigation measures were scaled down in September 2020, we extended these activities to communities to raise further awareness about tuberculosis and COVID-19 and to encourage people to seek care when symptomatic. We developed a standardized guide to support these sensitization activities, focused on (i) raising awareness about tuberculosis symptoms and risks; (ii) educating individuals to request tuberculosis screening; and (iii) providing information about where tuberculosis screening and testing was offered.

**Household contact tracing**

To safely resume home visits, we provided community-based health-care workers with refresher training during lunchtime meetings, using standardized training guides. Trainings were led by staff of the health ministry and implementing partner organizations. Community-based health-care workers were provided with specific education on COVID-19 infection and prevention control measures and were also supplied with washable masks and hand sanitizer.

COVID-19: coronavirus disease 2019.

### Data analysis

We undertook an interrupted time-series analysis using an ordinary least-squares segmented linear regression model. We used the Cumby–Huizinga general test and autocorrelation plots (up to lag order = 12) to assess for autocorrelation and possible seasonality. We used Newey–West standard errors to account for autocorrelation. The user-written ITSA command within Stata version 17.0 (Stata Corp., College Station, United States of America) was used to conduct interrupted time-series analyses.^13^

We defined three time periods for all interrupted time-series analyses: before the pandemic (Period 1, January 2019 to February 2020); during the pandemic, at the start of pandemic mitigation measures (Period 2, April 2020 to June 2020); during the pandemic, after implementation of tuberculosis case-finding measures (Period 3, August 2020 to September 2021). March 2020 and July 2020 represented phase-in periods and were not included in the data analysis. For March, the first COVID-19 cases were announced on 18 March 2020 and transmission mitigation measures were put into place later that month. For July, the scale-up of active tuberculosis case-finding activities across provinces did not begin until later in the month. Therefore, the effects of such measures on tuberculosis notifications in March and July, respectively, may not have been apparent until the following complete calendar month. Thus, two interruptions occurred between February and April 2020 (interruption 1) and between June and August 2020 (interruption 2).

We estimated several pre- and post-interruption measures of monthly tuberculosis case notifications and corresponding 95% confidence intervals (CI), including: (i) baseline trends in tuberculosis notifications before the pandemic (Period 1 trend); (ii) the absolute number of tuberculosis notifications and the relative per cent difference (compared with the counterfactual) in notifications immediately following interruptions 1 and 2 (change in level); (iii) the absolute trend in tuberculosis notifications during Periods 2 and 3 and the trend in these periods relative to the previous period (change in trend for Periods 2 and 3).

We also estimated the predicted number of tuberculosis case notifications in September 2021 had the pandemic not occurred and had the subsequent tuberculosis response measures not been implemented (the counterfactual) and compared this with the number of tuberculosis case notifications predicted for September 2021 after accounting for both interruptions (that is, observed notifications). We conducted all analyses overall (at the national level) and according to age (≥15 years, < 15 years), HIV status (positive, negative), tuberculosis classification type (microbiologically confirmed pulmonary tuberculosis, clinically diagnosed pulmonary tuberculosis or extrapulmonary tuberculosis) and by province. We wanted to evaluate whether possible impacts on tuberculosis notifications differed by sex, but due to the way routine data are aggregated and reported in Zambia, monthly sex-disaggregated data were not available for interrupted time-series analyses. We therefore descriptively assessed quarterly sex-disaggregated notification data to evaluate whether differential impacts by sex may be present.

## Results

### Before pandemic 

**Fig. 2:**
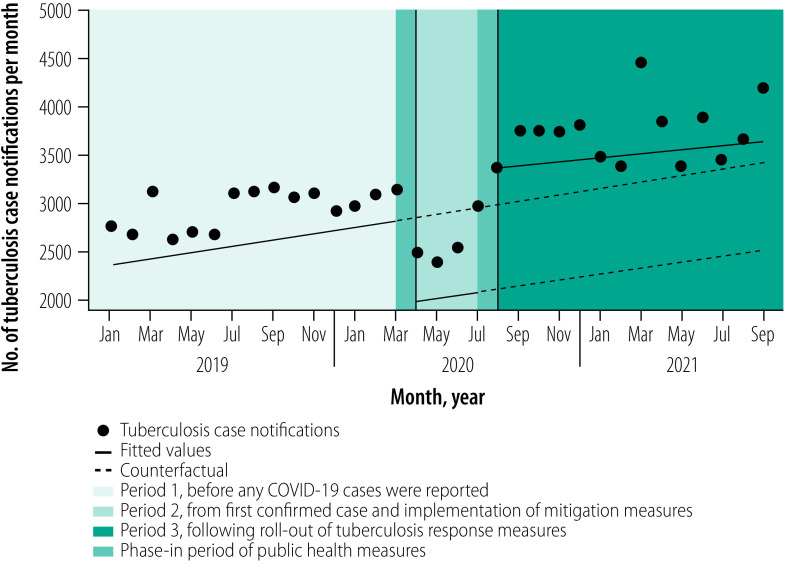
National level tuberculosis notifications among adults in Zambia, January 2019 to September 2021

**Fig. 3:**
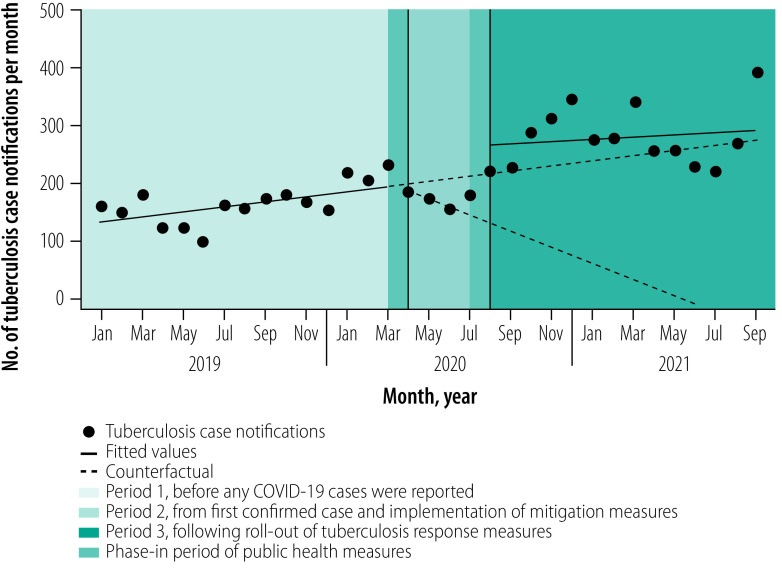
National level tuberculosis notifications among children in Zambia, January 2019 to September 2021

**Fig. 4:**
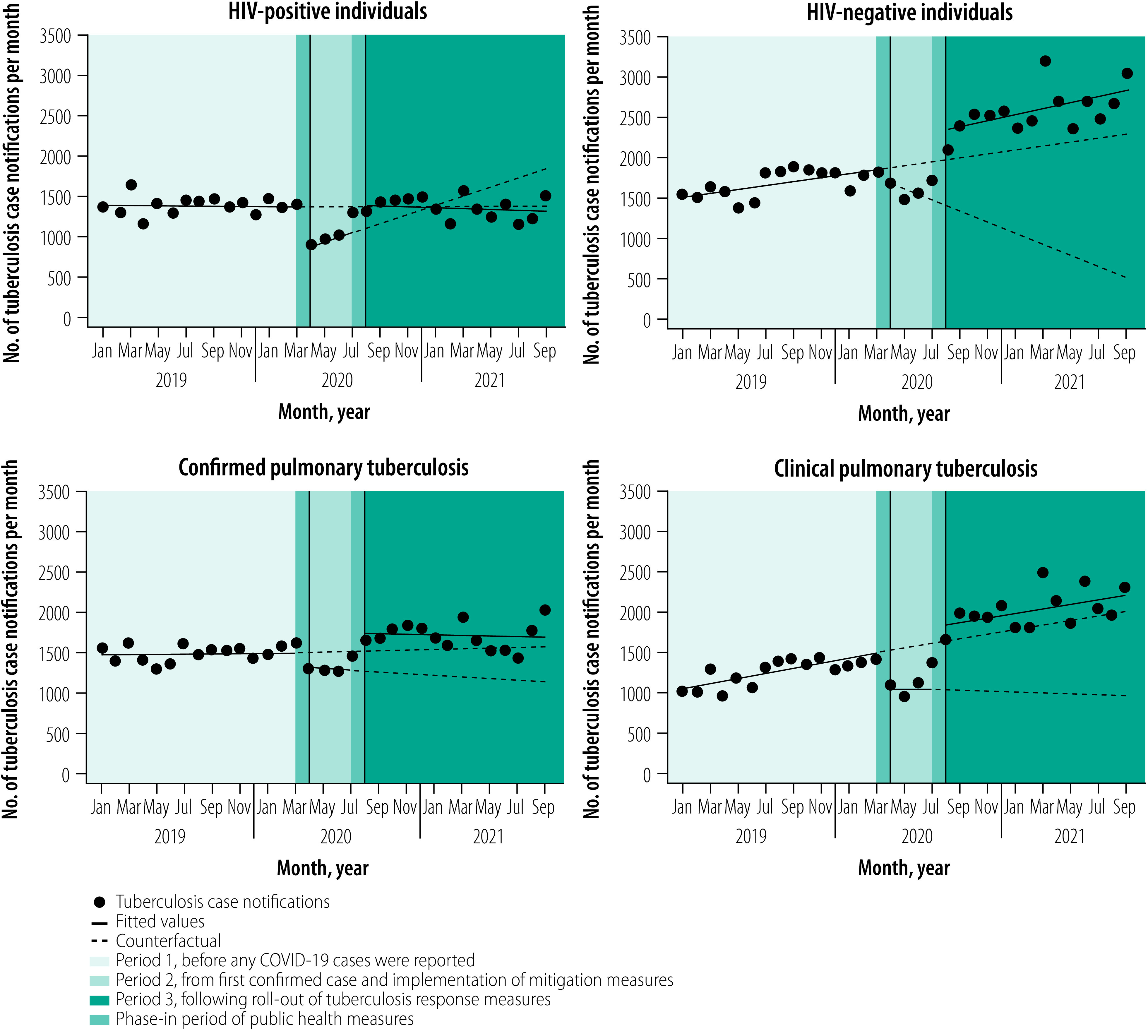
National level tuberculosis notifications among key subgroups in Zambia, January 2019 to September 2021

COVID-19: coronavirus disease 2019; HIV: human immunodeficiency virus.

Note: More details about the time periods and the pandemic mitigation measures are in the footnote to Fig. 1.

Before the pandemic started in Zambia, tuberculosis notifications were slowly increasing by about 32 cases (95% CI: 22 to 42) notified per month, from a baseline of approximately 2890 cases (95% CI: 2804 to 2975) in January 2019 to 3337 cases (95% CI: 3252 to 3421) in February 2020 (Table 1; Fig. 1). Although trends in monthly tuberculosis notifications varied across subgroups and provinces, notifications were not declining in any subgroup or province before COVID-19 cases were found in Zambia (Table 1; Fig. 2; Fig. 3; Fig. 4). 

**Table 1:** Impact of COVID-19 pandemic and implementation of tuberculosis case-finding activities on tuberculosis case notifications in Zambia, January 2019 to September 2021

Variable	Before pandemic, Period 1			After pandemic and mitigation measures, Period 2				After tuberculosis response measures, Period 3		
	Jan 2019	Jan 2019–Feb 2020		April 2020		Apr 2020–Jun 2020		Aug 2020		Aug 2020–Sep 2021
	Initial no. of cases (95% CI)	Monthly trend in no. of cases (95% CI)		Immediate change in no. of cases (95% CI)	% difference (95% CI)	Monthly trend in no. of cases (95% CI)		Immediate change in no. of cases (95% CI)	% difference (95% CI)	Monthly trend in no. of cases (95% CI)
**Overall**	2890 (2804 to 2975)	32 (22 to 42)		−733 (−831 to −634)	−22 (−24 to −19)	8 (−23 to 39)		1206 (1038 to 1375)	45 (38 to 51)	20 (−3 to 44)
**Age, years**										
≥15	2756 (2672 to 2840)	28 (17 to 39)		−722 (−836 to −609)	−22 (−25 to −20)	22 (−10 to 53)		1074 (929 to 1218)	42 (36 to 48)	18 (0 to 36)
< 15	134 (101 to 166)	4 (0 to 8)		−11 (−45 to 23)	−5 (−20 to 9)	−14 (−15 to −12)		133 (79 to 186)	88 (54 to 100)	2 (−6 to 11)
**HIV status**										
Positive	1376 (1279 to 1472)	−1 (−7 to 5)		−493 (−532 to −453)	−36 (−38 to −35)	54 (52 to 55)		308 (254 to 363)	30 (25 to 35)	−7 (−15 to 1)
Negative	1524 (1443 to 1605)	24 (12 to 36)		−224 (−353 to −94.5)	−12 (−17 to −6)	−69 (−100 to −37)		966 (809 to 1122)	65 (53 to 77)	37 (19 to 56)
**Tuberculosis type**										
Pulmonary tuberculosis, confirmed	1494 (1377 to 1611)	4 (−8 to 16)		−193 (−286 to −101)	−12 (−17 to −8)	−14 (−15 to −12)		457 (330 to 583)	35 (26 to 44)	−3 (−30 to 25)
Pulmonary tuberculosis, clinical	1047 (991 to 1103)	31 (23 to 39)		−444 (−528 to −360)	−29 (−33 to −25)	−3 (−29 to 23)		794 (683 to 906)	73 (60 to 85)	27 (17 to 38)
Extrapulmonary tuberculosis	349 (324 to 374)	−3 (−6 to 1)		−95 (−141 to −50)	−31 (−42 to −21)	25 (11 to 38)		−45 (−99 to 9)	−16 (−33 to 1)	−5 (−10 to 0)
**Province**										
Central	164 (143 to 186)	0 (−2 to 3)		−37 (−56 to −18)	−22 (−31 to −14)	18 (11 to 25)		87 (48 to 126)	48 (25 to 70)	3 (−1 to 7)
Copperbelt	631 (570 to 692)	11 (4 to 18)		−142 (−235 to −48)	−18 (−27 to −8)	19 (−9 to 47)		381 (203 to 559)	53 (26 to 79)	0 (−13 to 14)
Eastern	131 (114 to 147)	−1 (−3 to 1)		18 (−12 to 48)	16 (−13 to 46)	−14 (−20 to −7)		53 (30 to 77)	60 (28 to 92)	2 (0 to 5)
Luapula	158 (−151 to 166)	1 (0 to 2)		10 (−1 to 21)	6 (0 to 12)	−12 (−16 to −7)		54 (31 to 77)	37 (21 to 54)	7 (5 to 9)
Lusaka	1095 (931 to 1258)	5 (−12 to 22)		−375 (−471 to −279)	−32 (−37 to −27)	22 (8 to 35)		245 (117 to 372)	28 (14 to 43)	5 (42 to 85)
Muchinga	76 (62 to 90)	0 (−2 to 1)		−16 (−34 to 3)	−22 (−42 to −2)	2 (−1 to 5)		20 (−6 to 46)	33 (−8 to 74)	−1 (−4 to 2)
Northern	150 (127 to 173)	6 (3 to 9)		−35 (−65 to −4)	−14 (−24 to −4)	−10 (−15 to −5)		131 (103 to 159)	72 (56 to 89)	5 (1 to 8)
Northwestern	138 (130 to 146)	2 (1 to 4)		−41 (−58 to −23)	−23 (−31 to −15)	−5 (−12 to 2)		52 (9 to 95)	44 (8 to 79.6)	0 (−6 to 5)
Southern	197 (182 to 211)	3 (2 to 5)		−75 (89 to −62)	−30 (−34 to −26)	−2 (−7 to 4)		132 (94 to 171)	78 (53 to 100)	−1 (−5 to 3)
Western	150 (138 to 163)	5 (2 to 8)		−41 (−74 to −7)	−18 (−29 to −7)	−11 (−16 to −5)		51 (14 to 89)	33 (8 to 57)	1 (−4 to 6)

CI: confidence interval; COVID-19: coronavirus disease 2019; HIV: human immunodeficiency virus.

Notes: We analysed data from nationally aggregated, facility-level routine tuberculosis notification data. Period 1 was before any COVID-19 cases were reported. Period 2 was immediately following confirmation of COVID-19 cases and implementation of national pandemic mitigation measures. Period 3 was after roll-out of tuberculosis response measures across provinces, including tuberculosis active case-finding activities. March 2020 and July 2020 represented phase-in periods and were not included in the data analysis. No. of cases is the number of tuberculosis case notifications. Percentage difference is the relative difference in tuberculosis notifications immediately following a time interruption compared with the counterfactual. The data underpinning the calculations are visually represented in Fig. 1, Fig. 2, Fig. 3 and Fig. 4, where the observed number of tuberculosis notifications in the first month immediately following a time interruption are compared against the predicted counterfactual number of tuberculosis notifications in that same month assuming that there was no change in the trend of monthly tuberculosis notifications from the previous period.

COVID-19: coronavirus disease 2019.

Note: More details about the time periods and the pandemic mitigation measures are in the footnote to Fig. 1.

COVID-19: coronavirus disease 2019.

Note: More details about the time periods and the pandemic mitigation measures are in the footnote to Fig. 1.

### After implementation of measures

The relationship between daily COVID-19 cases and monthly tuberculosis notifications is shown in Fig. 1. In April 2020, following the confirmation of COVID-19 in Zambia and implementation of transmission mitigation measures the previous month, the overall number of tuberculosis notifications fell from a predicted 3400 cases (95% CI: 3298 to 3503) to an observed 2668 cases (95% CI: 2628 to 2707), an immediate decline of −733 cases (95% CI: −831 to −634). The decrease represented a −22% (95% CI: −24 to −19) decline nationally relative to the counterfactual (Table 1). Immediate declines in tuberculosis notifications were largely driven by reductions in clinically diagnosed pulmonary tuberculosis cases (−29%; 95% CI: −33 to −25) and extrapulmonary tuberculosis cases (−31%; 95% CI: −42 to −21) compared with a smaller immediate decline (−12%; 95% CI: −17 to −8) in microbiologically confirmed tuberculosis cases. The largest immediate impact in notifications across subgroups was −36% (95% CI: −38 to −35) among people living with HIV compared with −12% (95% CI: −17 to −6) among HIV-negative individuals (Table 1; Fig. 4). Tuberculosis notifications among children were less immediately impacted than among adults (Table 1; Fig. 2; Fig. 3). The immediate impact of pandemic measures differed substantially at the provincial level, with significant negative impacts observed in eight of 10 provinces. Lusaka (−32%; 95% CI: −37 to −27) and Southern provinces (−30%; 95% CI: −34 to −26) reported the largest immediate declines in tuberculosis notifications.

In the three months following the start of the pandemic and after a large initial decline in national tuberculosis notifications, notifications remained steady on a month-to-month basis and did not substantially differ from pre-pandemic monthly trends (Table 1; Fig. 1). Following large immediate declines in tuberculosis notifications among people living with HIV, the monthly trend in notifications increased compared with pre-pandemic trends (Table 1; Fig. 4). In contrast, after a smaller immediate decline among HIV-negative individuals, monthly tuberculosis notifications decreased substantially compared with pre-pandemic trends. Significant declines in month-to-month tuberculosis notification trends compared with pre-pandemic levels were observed among children, for microbiologically confirmed tuberculosis cases and across several provinces.

### After tuberculosis response

In July 2020, the National Tuberculosis and Leprosy Programme began rolling out several tuberculosis response measures in all provinces to bolster tuberculosis detection. The following month (August 2020), national tuberculosis notifications immediately increased from a predicted 2700 cases (95% CI: 2603 to 2796) to an observed 3906 cases (95% CI: 3761 to 4051), a rise of 1206 cases (95% CI: 1038 to 1375) and a 45% (95% CI: 38 to 51) increase (Table 1). Immediate increases in tuberculosis notifications were observed across nearly all subgroups, except for extrapulmonary tuberculosis cases (Fig. 2; Fig. 3; Fig. 4). The relative increase in tuberculosis notifications was more pronounced among children (88%; 95% CI: 54 to 100) than adults (42%; 95% CI: 36 to 48); among HIV-negative (65%; 95% CI: 53 to 77) than HIV-positive people (30%; 95% CI: 25 to 35); and for clinically diagnosed tuberculosis cases (73%; 95% CI: 60 to 85) compared with microbiologically confirmed cases (35%; 95% CI: 26 to 44). Immediate increases in tuberculosis notifications in August 2020 were observed in all 10 provinces but with a differential impact (range: 28% to 78%).

The trend in monthly national tuberculosis notifications between August 2020 and September 2021 remained steady (20; 95% CI: −3 to 44; Table 1). Stable or small increases in monthly tuberculosis notification trends during this period was observed across all subgroups and provinces. The proportional distribution of tuberculosis notifications according to sex did not significantly differ across each of the three periods between January 2019 and September 2021 (range of total proportion of notifications among males: 64% to 68%; Table 2).

**Table 2:** Quarterly national tuberculosis notifications in Zambia disaggregated by sex, January 2019 to September 2021

Reporting quarter by year	No. of tuberculosis notifications				% of notifications in males (95% CI)
	Total	Males	Females		
**2019**					
Q1	9 044	5 966	3 078		66 (65 to 67)
Q2	8 348	5 439	2 909		65 (64 to 66)
Q3	9 876	6 404	3 472		65 (64 to 66)
Q4	9 598	6 146	3 452		64 (63 to 65)
**2020**					
Q1	9 863	6 382	3 481		65 (64 to 66)
Q2	7 898	5 055	2 843		64 (63 to 65)
Q3	10 515	7 129	3 386		68 (67 to 69)
Q4	12 250	7 983	4 267		65 (64 to 66)
**2021**					
Q1	11 769	7 722	4 047		66 (65 to 66)
Q2	11 432	7 406	4 026		65 (64 to 66)
Q3	11 684	7 558	4 126		65 (64 to 66)

CI: confidence interval; Q: quarter.

Note: Sex-disaggregated data on monthly tuberculosis notifications were not available due to how routine notification data are collected and reported, and could therefore not be reported in the same manner as the primary interrupted time-series analyses.

In September 2021, the overall number of tuberculosis notifications was 4107 cases (95% CI: 3923 to 4292). This figure was not significantly different to the estimated number of notifications assuming a continuation of pre-pandemic trends (difference: 229; 95% CI: −100 to 558; Table 3; Fig. 1). The number of tuberculosis notifications in September 2021 in each subgroup and in nine of 10 provinces were similar to or exceeded the number of tuberculosis notifications predicted for September 2021, assuming a continuation of pre-pandemic trends.

**Table 3:** Comparison of predicted and actual number of monthly tuberculosis case notifications in Zambia by key subgroups and provinces, September 2021

Variable	Estimated no. of monthly tuberculosis case notifications (95% CI)		
	Before pandemic, counterfactual	After pandemic and tuberculosis response measures	Difference
**Overall**	3879 (3 631 to 4 126)	4107 (3 923 to 4 292)	229 (−100 to 558)
**Age, years**			
≥15	3613 (3 335 to 3 892)	3819 (3 672 to 3 965)	205 (−129 to 540)
< 15	265 (173 to 358)	289 (215 to 363)	23 (−98 to 145)
**HIV status**			
Positive	1342 (1 218 to 1 468)	1304 (1 236 to 1 373)	−39 (−188 to 111)
Negative	2266 (1949 to 2 583)	2814 (2 673 to 2 955)	548 (173 to 923)
**Tuberculosis type**			
Pulmonary tuberculosis, confirmed	1603 (1 320 to 1 887)	1724 (1 467 to 1980)	120 (−271 to 511)
Pulmonary tuberculosis, clinical	2011 (1 812 to 2 211)	2176 (2092 to 2 261)	165 (−69 to 399)
Extrapulmonary tuberculosis	264 (165 to 363)	208 (164 to 252)	−56 (−167 to 55)
**Province**			
Central	170 (111 to 228)	321 (298 to 343)	151 (83 to 219)
Copperbelt	972 (783 to 1 160)	1104 (1 036 to 1 173)	132 (−75 to 340)
Eastern	94 (33 to 155)	163 (136 to 189)	69 (0 to 137)
Luapula	180 (156 to 203)	278 (266 to 291)	99 (72 to 125)
Lusaka	1251 (896 to 1 607)	1181 (999 to 1 364)	−70 (−483 to 344)
Muchinga	66 (53 to 90)	72 (53 to 90)	5 (−45 to 56)
Northern	336 (246 to 427)	349 (320 to 377)	12 (−86 to 110)
Northwestern	210 (175 to 244)	161 (120 to 202)	−48 (−101 to 4)
Southern	300 (261 to 340)	283 (254 to 312)	−17 (−67 to 33)
Western	299 (217 to 382)	194 (153 to 236)	−105 (−200 to −10)

CI: confidence interval; COVID-19: coronavirus disease 2019; HIV: human immunodeficiency virus.

Note: We estimated the predicted number of tuberculosis case notifications in September 2021 had COVID-19 not occurred and had the subsequent tuberculosis response measures not been implemented (the counterfactual) and compared this with the number of tuberculosis case notifications predicted for September 2021 based on observed data. Before pandemic period was January 2019–February 2020. After pandemic and tuberculosis response period was April 2020–June 2020 and August 2020–September 2021, respectively.

## Discussion

We found that national tuberculosis notifications in Zambia immediately declined by 22% following the confirmation of COVID-19 cases and implementation of mitigation measures designed to stem further COVID-19 transmission. There were substantial differences in the immediate and subsequent impact of the pandemic on tuberculosis notifications among subgroups and across provinces in Zambia. The immediate effect was most pronounced among people living with HIV, who had threefold higher declines than HIV-negative individuals, and in Lusaka province, which accounts for more than 40% of tuberculosis notifications in Zambia.^7^ Following the initial scale-up of several tuberculosis response measures in July 2020 tuberculosis notifications immediately increased by 45%. Notifications then remained stable over the subsequent months, despite two additional, larger surges of COVID-19, and were similar to pre-pandemic levels in September 2021. These data highlight the importance of careful and continued surveillance of important public health problems during the COVID-19 and future pandemics. The results point to the feasibility and positive impact associated with implementing coordinated public health responses to alleviate the detrimental effect of the pandemic. Given the success associated with the activities implemented, such initiatives will be continued as part of Zambia’s national tuberculosis programmatic strategy.

Studies in several high-tuberculosis burden countries in sub-Saharan Africa^14^^–^^16^ and other parts of the world^17^^,^^18^ also found an immediate detrimental impact of the pandemic and associated mitigation measures on tuberculosis diagnostic and treatment outcomes. The impact on national-level tuberculosis notifications in Zambia (22%) is slightly lower than what was reported from facilities in Nigeria (34%)^15^ and Uganda (43%).^14^ However, we found substantial differences in the effects of the pandemic on tuberculosis notifications in Zambia that ranged from no immediate impact in some provinces to immediate adverse impacts exceeding 30% in others. The reasons underpinning such heterogeneity may in part reflect urban versus rural differences. People in more rural settings may have perceived themselves at lower risk for COVID-19 and thus their health-seeking behaviours were less initially impacted. There may also be a differential impact of temporarily suspending community-based tuberculosis activities, including sensitization activities and household case-finding. 

We also found that immediate declines in tuberculosis notifications were more pronounced among people living with HIV compared with HIV-negative patients. However, this difference likely reflects far fewer people living with HIV attending facilities during the early stages of the COVID-19 pandemic. A national campaign had been initiated to make early contact with all people living with HIV and to provide extended ART refills (up to 6 months) in March and early April 2020.^12^ Notably, in the first 3 months of the pandemic in Zambia, and following a large immediate decline in tuberculosis notifications, we found that case numbers either stayed flat or continued to decline among most subgroups and in most provinces. These data have important implications, as even short disruptions in tuberculosis services and transient declines in tuberculosis notifications may result in thousands of additional tuberculosis-related deaths and many new incident tuberculosis cases due to prolonged periods of infectiousness. Such a result could reverse hard-won progress in tuberculosis care by several years.^1^^,^^2^^,^^4^^,^^14^

We implemented enhanced tuberculosis response activities, scaled up facility-based active case-finding measures, and increased access to improved tuberculosis diagnostic tools. These activities appeared to be associated with a marked improvement in tuberculosis notifications during the pandemic, and as the activities were sustained, so too was their positive impact on notifications as the pandemic continued. Due to our quasi-experimental study design, we cannot discern whether improvements in tuberculosis notifications are the sole result of these activities or may be due to other secular trends.^19^ For example, around the same time tuberculosis response measures were initially being scaled up, there may have been more individuals presenting to health facilities in the context of either greater population mobility due to defiance of COVID-19 transmission control measures or less fear of contracting COVID-19. However, this explanation seems unlikely given that COVID-19 cases were increasing in Zambia while active tuberculosis case-finding strategies were being rolled out. Notably, following the implementation of carefully coordinated steps to bolster tuberculosis diagnoses, there was a large, immediate increase in tuberculosis notifications. This positive effect was seen across nearly all subgroups and provinces and was directly preceded by relatively stable tuberculosis notification trends. Collectively, this provides compelling evidence that the implementation of tuberculosis response measures was responsible for increasing tuberculosis notification rates to pre-pandemic levels.

The strengths of this analysis include the use of routine, national-level programmatic data and of disaggregated analyses among several key subgroups and in all Zambian provinces. This method allowed us to assess for possible differential trends in tuberculosis notifications before and during the COVID-19 pandemic. Furthermore, we analysed notification trends across three periods covering 33 months, including multiple waves of the pandemic, which allowed us to determine that gains in notifications following implementation of tuberculosis response activities were sustained. There were some limitations, however. We were unable to determine what factors underpinned the immediate decrease in tuberculosis notifications in Zambia following the emergence of COVID-19 cases. However, the change likely reflects both individual- and health-system-related factors. For example, individuals may have been reluctant to seek care for their symptoms due to fear or stigma.^20^ Additionally, during the early pandemic, some laboratory technicians refused to process specimens over safety concerns. This issue was resolved after providing education on COVID-19 infection control measures and additional personal protective equipment. Under-notification and underreporting may have also contributed to declines in tuberculosis notifications during this period. Finally, due to the prolonged nature of tuberculosis treatment, coupled with the time required for reporting and aggregation of treatment data, we were unable to assess the impact of the pandemic on tuberculosis treatment completion rates. It will be important to monitor and formally evaluate for any detrimental effects of the pandemic on tuberculosis treatment completion rates.

In conclusion, the COVID-19 pandemic and the associated mitigation measures had a substantial impact on tuberculosis case notifications in Zambia. A carefully coordinated public health response, including active tuberculosis case-finding strategies, was feasible to implement and was associated with a return of tuberculosis case notifications to pre-pandemic levels. The gains were sustained throughout subsequent waves of the pandemic. Continued vigilance will be required during the ongoing pandemic to ensure high tuberculosis diagnosis and treatment coverage levels.
